# Integracides: Tetracyclic Triterpenoids from *Fusarium* sp.—Their 5-Lipoxygenase Inhibitory Potential and Structure–Activity Relation Using In Vitro and Molecular Docking Studies

**DOI:** 10.3390/life12122095

**Published:** 2022-12-13

**Authors:** Maan T. Khayat, Khadijah A. Mohammad, Gamal A. Mohamed, Martin K. Safo, Sabrin R. M. Ibrahim

**Affiliations:** 1 Department of Pharmaceutical Chemistry, Faculty of Pharmacy, King Abdulaziz University, Jeddah 21589, Saudi Arabia; 2 Department of Natural Products and Alternative Medicine, Faculty of Pharmacy, King Abdulaziz University, Jeddah 21589, Saudi Arabia; 3 Department of Medicinal Chemistry, School of Pharmacy, Virginia Commonwealth University, Richmond, VA 23219, USA; 4 Department of Chemistry, Preparatory Year Program, Batterjee Medical College, Jeddah 21442, Saudi Arabia; 5 Department of Pharmacognosy, Faculty of Pharmacy, Assiut University, Assiut 71526, Egypt

**Keywords:** *Fusarium* sp., integracides, triterpenoids, 5-lipoxygenase, inflammation, drug discovery, molecular docking

## Abstract

Inflammation is a complicated disorder that is produced as a result of consecutive processes. 5-LOX (5-lipoxygenase) is accountable for various inflammation mediators and leukotrienes synthesis, and its inhibition is the target of anti-inflammation therapeutics. Fungi have acquired enormous attentiveness because of their capability to biosynthesize novel bio-metabolites that reveal diversified bio-activities. A new tetracyclic triterpenoid, integracide L (**1**), along with integracides B (**2**) and F (**3**), were separated from *Mentha longifolia*-associated *Fusarium* sp. (FS No. MAR2014). Their structures were verified utilizing varied spectral analyses. The isolated metabolites (**1**–**3**), alongside the earlier reported integracides G (**4**), H (**5**), and J (**6**), were inspected for 5-LOX inhibition capacity. Interestingly, **1**–**6** possessed marked 5-LOX inhibition potentials with IC_50_s ranging from 1.18 to 3.97 μM compared to zileuton (IC_50_ 1.17 µM). Additionally, molecular docking was executed to examine the interaction among these metabolites and 5-LOX, as well as to validate the in vitro findings. The docking study revealed their inhibitory activity interactions in the binding pocket. These findings highlighted the potential of integracides as lead metabolites for anti-inflammation drug discovery.

## 1. Introduction

Inflammation is a complicated reaction that is controlled by an interaction between the cells and inflammatory mediators [1]. Among the most important enzymes involved in this process are cyclo-oxygenases (COX-1 and 2), phospholipase A2, and lipoxygenases (LOXs) enzymes [2,3]. Leukotrienes (LTs) represent potent lipid mediators of allergic and inflammatory reactions such as kidney, skin, cardiovascular, allergic diseases, arthritis, asthma, cancer, neurodegenerative disorders, and metabolic syndrome [4,5]. The 5-LOX metallo-enzyme activates the conversion of arachidonic acid (AA) to leukotrienes (LTs) [6,7]. Thus, the inhibition of the 5-LOX pathway is known to be interesting for treating various inflammatory disorders. 5-LOX inhibitors are of potential therapeutic value. Minocyclines and zileuton are LOX inhibitors available in pharmacies; however, their uses have been limited or forbidden due to their serious side effects [8,9]. Due to such side-effects, the development of new LOX inhibitors with minimum side effects is a recent challenge [10].

Fungi have acquired enormous attentiveness because of their capability to biosynthesize novel bio-metabolites possessing diversified bio-properties that are utilized in pharmaceutical, medicinal, and agricultural fields [11,12,13,14,15,16,17,18]. Endophytic fungi can inhabit the internal living tissues of the host plants, often without producing any external symptoms or apparent negative effects [19,20,21,22]. They are a prominent pool of new and biologically active natural products such as terpenoids, alkaloids, steroids, isocoumarins, quinones, lignans, phenols, phenylpropanoids, and lactones [23,24,25,26,27,28]. The *Fusarium* species, a widespread cosmopolitan group of fungi, have been separated from both marine and terrestrial sources [20,29,30]. Integracides, an under-explored class of oxygenated tetracyclic 4,4-dimethylergostane triterpenoids, possessing a 12-acetyl-Δ^8,14^-diene-11-ol moiety have been reported only from *Fusarium* sp. [20,31,32,33,34,35]. Integracides were reported to possess diversified bio-activities: cytotoxic, anti-leishmanial, HIV-1 integrase, elastase, rhinovirus 3C protease, and cholesteryl ester transfer protein inhibitory [20,30,31,32]. Integracides could represent a potential therapeutic target for degenerative and inflammatory diseases via their elastase inhibitory potential [33]. Our earlier studies on *Fusarium* sp. (FS No. MAR2014) led to the characterization of 4 integracides [20,30]. When seeking more integracides from this fungus, a new culture of *Fusarium* sp. (FS No. MAR2014) was inspected. Chromatographic separation of the EtOAc extract yielded a new integracide derivative: integracide L (**1**), along with three known metabolites. Their chemical structures were assigned by extensive spectral analyses and were compared with the literature. Additionally, the 5-lipoxygenase inhibition (5-LOI) capacity of integracides L (**1**), B (**2**), and F (**3**) and the formerly separated integracides G (**4**), H (**5**), and J (**6**) were assessed [20,30]. Molecular docking was also carried out for these metabolites to verify their 5-LOI potential. In addition, the structure–activity relationship of these metabolites was discussed.

## 2. Results

### 2.1. Purification of Metabolites

**Figure 1:**
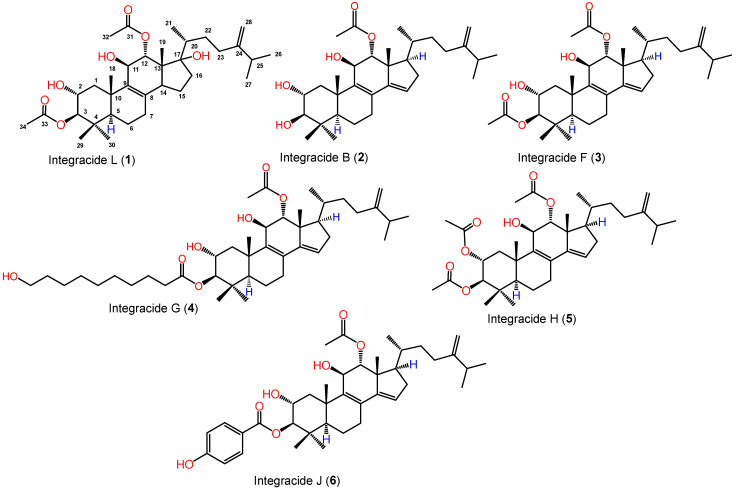
Chemical structures of integracides **1**–**6** isolated from *Fusarium* sp. (FS No. MAR2014).

From the *Fusarium* sp. (FS No. MAR2014) MeOH extract, a new integracide derivative integracide L (**1**) and two known metabolites (**2** and **3**) were purified utilizing SiO_2_/Sephadex LH-20/RP-18 (Figure 1). The known metabolites were specified as integracides B (**2**) [20,30] and F (**3**) [20,30] by comparing their spectral data to earlier published data and co-TLC with authentic samples (Supplementary Material).

### 2.2. Structural Elucidation of Integracide L

**Figure 2:**
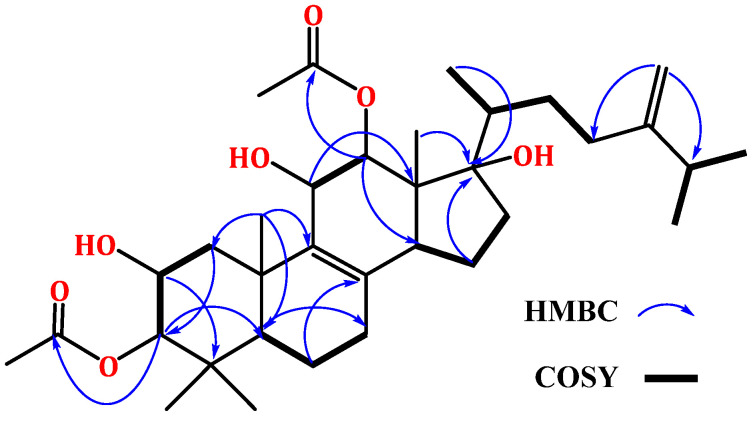
Some key ^1^H-^1^H COSY and HMBC correlations of **1**.

Compound **1** was separated as a white amorphous powder. It had a C_34_H_54_O_7_ molecular formula based on the molecular peak at *m*/*z* 575.3954 [M + H]^+^ (calcd for C_34_H_55_O_7_, 575.3948) in HRESIMS, indicating eight DBE. It showed a UV band at λ*_max_* 235 nm. The IR displayed absorptions at 3439, 1729, 1663, and 879 cm^−1^, corresponding to hydroxyl, ester carbonyl, and the exocyclic *di*-substituted double bond, respectively. Compound **1** was one degree of unsaturation and 18 mass units more than integracide F (**3**), indicating the loss of one double bond and the presence of an extra OH group in **1**. It exhibited characteristic HRESIMS fragments at *m*/*z* 532.3758 [M + H-OAc]^+^ and 490.3658 [M + H-2OAc]. It’s NMR data of **1** were similar to that of **3** [20]. Thirty-four carbons were noted in the HSQC and ^13^C spectra, consisting of seven methyls, eight methylenes, and eight methines, four of them for oxymethine carbons, and nine quaternary carbons, including three olefinic, two carbonyls, and an oxygen-bonded carbon. The ^1^H and ^13^C NMR spectra displayed signals at δ_H_ 4.66 and 4.70 (H-28)/δ_C_ 106.9 (C-28) and 155.4 (C-24), 127.4 (C-8), and 143.5 (C-9) indicating the existence of a *tetra*-substituted olefinic double bond and an exomethylene group in **1** (Table 1). Their position at C_8_-C_9_ and C_24_-C−_28_ was assured by the observed HMBC peaks of H-26/H-22/H-27/C-24, H-28/C-23, and C-25, H-23/C-24, and C-28, H-6/H-11/H-15/C-8, and H-7/H-12/H-18/C-9 (Figure 2). Four methyls singlets at δ_H_ 1.20 (H-18), 0.91 (H-30), 0.73 (H-29), and 0.61 (H-19) and methyl doublet at δ_H_ 1.22 (H-21), relating to the carbons at δ_C_ 22.1 (C-18), 28.5 (C-30), 17.9 (C-29), and 13.2 (C-19) in the HSQC were observed. The HMBC peaks from H-18/C-1, C-9, C-5, and C-10, H-21/C-22 and C-17, H-30 and H-29/C-4, C-3, and C-5, and H-19/C-17, C-13, C-12, and C-14 proved the location of the CH_3_ groups. The ^1^H-^1^H COSY spin system extended from H-14 to H-16 and indicated the absence of the C_14_-C_15_ *tri*-substituted olefinic double bond in **1**. Moreover, the ^1^H and ^13^C displayed signals that were characteristic of an isopropyl moiety, including a multiplet methine at δ_H_ 2.19 (H-25)/32.3 (C-25) and two methyls doublet at δ_H_ 0.98 (H-26)/δ_C_ 21.6 (C-26) and 0.96 (H-27)/δ_C_ 21.5 (C-27). This was secured by the ^1^H-^1^H COSY cross peaks of H-25/H-27 and H-26 and the HMBC peaks of H-25/C-26 and C-27 and H-26 and H-27/C-25. Its connectivity at C-24 was confirmed by the observed cross peaks of H-26 and H-27/C-24, H-28/C-25, and H-25/C-24 and C-23 in the HMBC. Furthermore, four oxymethine signals at δ_H_ 5.00 (H-12)/4.25 (H-11)/3.73 (H-3)/3.62 (H-3), correlating to the carbons at δ_C_ 78.3, 67.9, 66.8, and 88.0, respectively in the HSQC were observed. Their presence at C-12, C-11, C-2, and C-3, respectively, was established by the observed HMBC and ^1^H-^1^H COSY cross peaks (Figure 2).

A signal for the oxygen-linked quaternary carbon was observed at δ_C_ 86.0 (C-17). Its placement was secured by HMBC peaks of H-15, H-19, and H-12 to C-17. Moreover, the signals at δ_H_ 1.98 (H-32)/δ_C_ 20.6 (C-32), 2.05 (H-34)/21.0 (C-34), 169.5 (C-31), and 170.1 (C-33) indicated the existence of two acetoxy groups in **2** (Table 1).

**Figure 3:**
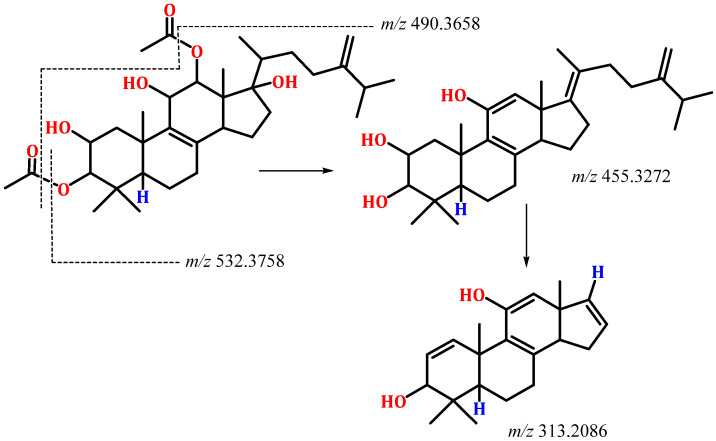
Possible fragmentation pattern of integracide L (**1**).

This was confirmed by the HRESIMS fragment peaks at *m*/*z* 532.3758 [M + H-OAc]^+^ and 490.3658 [M + H-2OAc] (Figure 3).

Their attachment at C-3 and C-12 was secured by the HMBC correlations of H-3/C-33 and H-12/C-31. Furthermore, three OH signals were noted at δ_H_ 5.21 (d, *J* = 6.8 Hz, 11-OH), 5.30 (brs, 2-OH), and 5.39 (s, 17-OH). The HMBC peaks of 11-OH/C-9/C-11/C-12, 2-OH/C-1/C-2/C-3, and 17-OH/C-13/C-17 proved their assignment. The relative configuration of **1** was assigned by comparing the ^1^H and ^13^C shifts and coupling constants with **3** and the related analogs [20,30,31,32]. Based on these findings, the structure of **1** was unambiguously elucidated and named integracide L.

### 2.3. 5-LOX Inhibitory Activity

**Figure 4:**
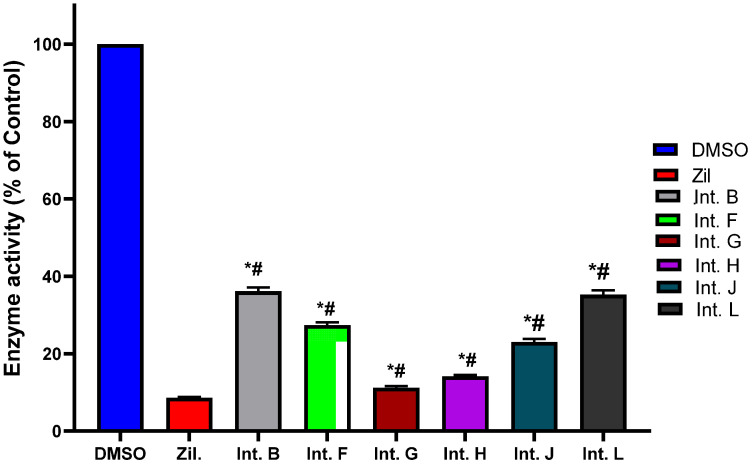
5-Lipoxygenase inhibition potential of integracides (**1**–**6**). * Compared to control group; # compared to indomethacin group (one-way ANOVA followed by Tukey–Kramer).

In the present investigation, integracides **1**–**6** reported from *Fusarium* sp. (FS No. MAR2014) were investigated to explore their 5-LOI capability. It is noteworthy that integracides G (**4**), H (**5**), and J (**6**) displayed potent 5-LOI potential (IC_50_s 1.18, 1.46, and 2.01 µM, respectively) compared to zileuton (IC_50_ 1.17 µM) (Figure 4). In addition, integracides L (**1**), B (**2**), and F (**3**) demonstrated significant effectiveness with IC_50_s, 3.45, 3.97, and 2.76 µM, respectively.

### 2.4. Molecular Docking Studies

**Figure 5:**
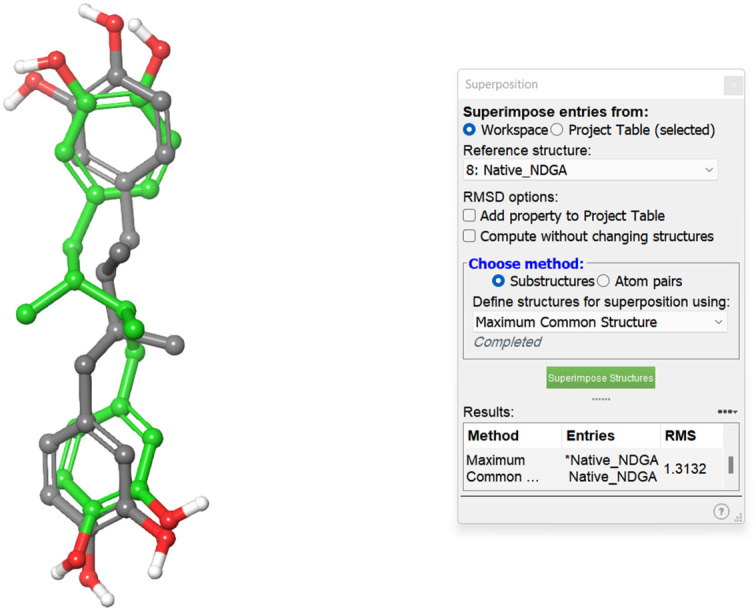
The 3D structures of the redocked NDGA superimposed on co-crystallized NDGA.

The docking study was performed with the Schrodinger program. Inregracides, zileuton, and NDGA (native co-crystallized-inhibitor) were prepared before docking using the “LigPrep” tool, where all ligand 2D structures were converted to 3D and energy minimized [36]. All the ligands’ possible tautomeric states and ionization were created as well. The crystal structure of stable 5-LOX complexed with the NDGA inhibitor was downloaded from the protein-data-bank (PDB-ID: 6N2W), prepared, and energy minimized employing “Protein_Preparation_Wizard” [37,38]. A grid box was created around the active site of the stable 5-LOX (PDB: 6N2W) containing the co-crystallized inhibitor NDGA using Glide’s “Receptor-Grid-Generation” tool in the Schrödinger suite. Finally, the “Ligand_Docking” tool was implemented for docking [39,40]. All the studied ligands were docked inside the grid box with XP (extra-precision) protocol [41]. The docking method was validated by redocking NDGA (native co-crystallized-inhibitor) in the prepared protein active site. When it was superimposed over the original co-crystallized inhibitor, it gave a 1.3132 Å RMSD (root-mean-square deviation) calculated value, indicating a valid docking method (Figure 5).

The chosen crystal structure of 5-LOX (PDB: 6N2W) is a protein-stabilized form in which stabilizing mutations are implemented, and membrane insertion loops are absent [37]. However, it still contains the canonical LOX fold, which is composed of the amino-terminal β-barrel membrane-binding domain and the α-helical domain in which the active site is located [38]. The active site of 5-LOX (where the arachidonic acid binds) forms a U-shape hydrophobic cavity, which contains a catalytic metal iron [37,42].

The docking findings performed with the XP mode for the energy-minimized 3D structures of integracides and zileuton are listed below in Table 2.

The docked compounds are ranked based on their gscores related to the free energy of the binding; the more negative scores imply better binding. The generated scores are the gscore (ranks different metabolites), emodel (ranks different conformers), and XP gscore. Glide employs emodel scoring to choose the docked compounds’ best poses; then, it ranks the best poses relying on the given gscores. The XP gscore ranks the Glide XP mode-created poses. In general, Glide uses the gscore to sort and rank the docked metabolites.

## 3. Discussion

Inflammation is a complicated response that is produced as a result of consecutive processes, one of which is arachidonic acid’s metabolism, which commences with oxidation by 5-LOX. It is proved that 5-LOX has a leading function in inflammation through the synthesis of various inflammation mediators and LTs (leukotrienes). 5-LOX has protruded as a prospective target for inflammation-linked disorders, including rheumatoid arthritis and asthma. Many of the available 5-LOX inhibitors are of synthetic origin and reveal untoward aftereffects, such as zileuton, that have the hepato-toxic potential [8,9]. Hence, finding out safe and efficacious anti-inflammation agents that modulate LT production is an imperious demand.

Fungi possess a miracle capability to produce unrivaled metabolites, and the varied bioactivities among them are terpenoids, including sesqui-, di-, mero-, and tri-terpenoids. Several reports stated the anti-inflammation potential of fungal terpenoids through varied mechanisms [43,44].

Among the reported fungal terpenoids, integracides are an uncommon class of tri-terpenoids that report mainly from *Fusarium* sp. (FS No. MAR2014). This class of metabolites displayed varied bioactivities.

In the current study, a new metabolite belonging to this class, along with the known ones, was purified and characterized utilizing various tools. Their in vitro anti-inflammation potencies, as assessed by their attenuation of 5-LOX, demonstrated the powerful 5-LOI capability of these metabolites. It was noted that compounds **4**, **5**, and **6** were the most potent compounds that had IC_50_ values that were comparable to that of the positive control, zileuton.

It is noteworthy that there were many studies that proved the anti-inflammatory effectiveness of triterpenoids via inhibition of 5-LOX [45,46,47].

In the molecular docking studies of this work, the data showed a better understanding of each inhibitor’s potency as well as their correlation with the bioassay throughout the binding and mode of interactions. For instance, the docking results of **4** were consistent with its biochemical inhibitory results, where it produced the lowest IC_50_ (1.18 μM) similar to that of zileuton (Figure 4).

The XP gscore ranking of integracide **F** (**3**), **H** (**5**), and J (**6**) showed minimal correlation to their in vitro inhibitory effectiveness, although the scores were close to each other. On the other hand, integracide **B** (**2**) and **L** (**1**) were ranked the least in terms of their gscores and IC_50_ values as well.

**Figure 6:**
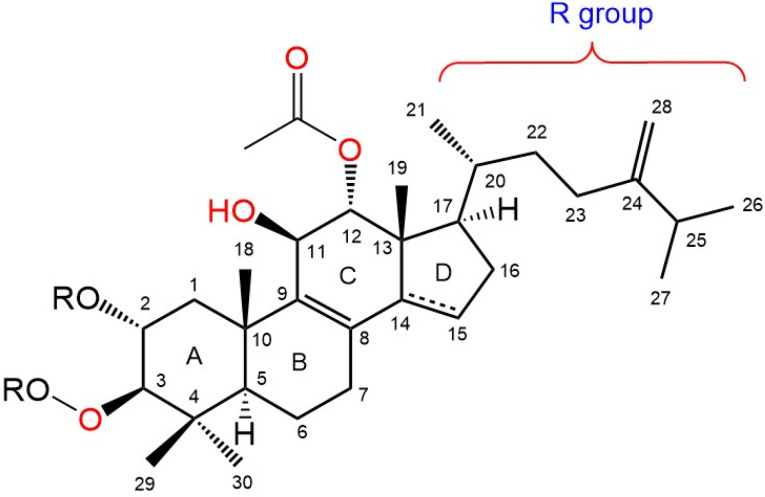
A representative integracide skeleton showing the numbering system.

The general schematic structure of integracides contains a cyclic hydrophobic skeleton composed of four rings (A, B, C, and D), a hydrophobic R group on C17 of ring D, an acetyloxy group on C12 of ring C, a hydroxyl group on C11 of ring C, a hydroxyl group on C2 of ring A (which may be acetylated) and a hydroxyl group on C3 of ring A (which is usually esterified by different groups) (Figure 6). The presence and type of the esterified groups on C3 could influence the binding affinity to the 5-LOX active site.

Integracide G (**4**) was the top-scored metabolite with a gscore of −6.708 kcal/mol among the other derivatives and was closer in score to NDGA (co-crystallized inhibitor) (Table 2).

**Figure 7:**
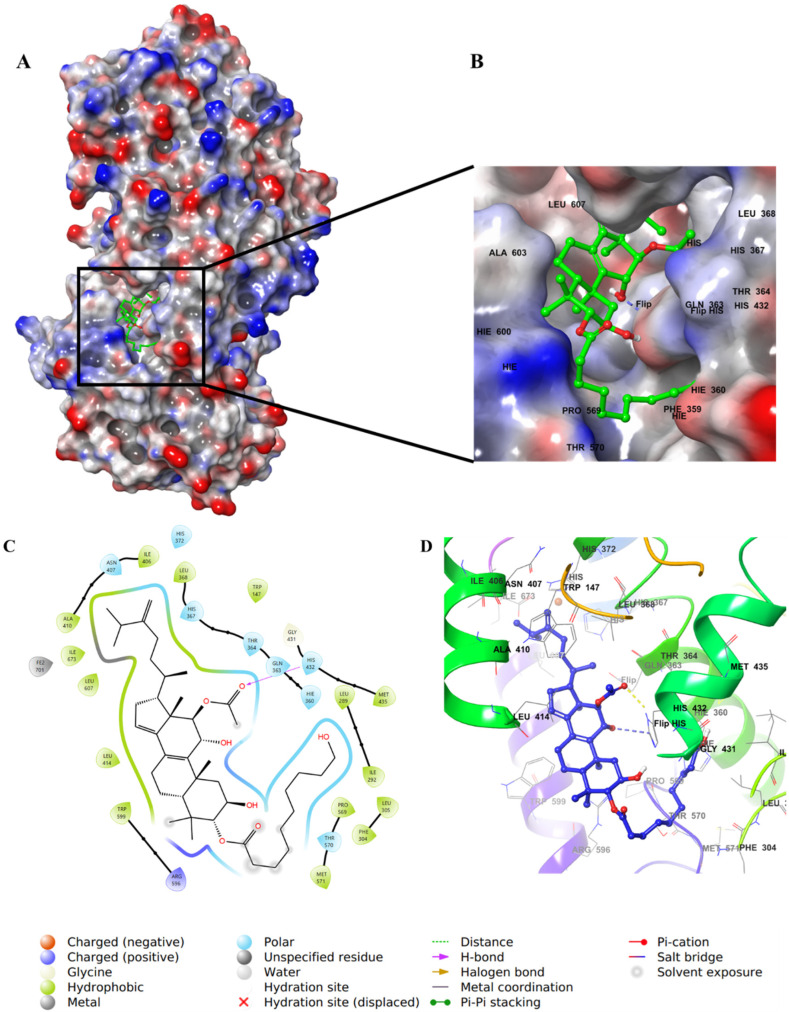
Molecular docking of integracide G (**4**) in stable human 5-LOX (PDB: 6N2W). (**A**) Molecular surface representation with solid style and electrostatic potential color scheme of red, white, and blue (min −0.3, max +0.3); (**B**) Zoomed look of integracide G (**4**) in the active site of 5-LOX in molecular surface; (**C**) 2D representation of binding interactions of integracide G with amino acid residues in the active site within 4 Å distance; (**D**) 3D representation of integracide G (**4**) in blue color within the active site of 5-LOX. The H–bond and aromatic–hydrogen interactions are in yellow and purple dotted lines, respectively.

The aliphatic substitution on the cyclopentene ring (ring D) involved in hydrophobic interactions with a hydrophobic pocket contained mainly Ile, Leu, and Ala residues. Both the OH group on C11 and the carbonyl oxygen of the acetyloxy group on ring C formed aromatic–hydrogen and H–bond interactions, respectively, with His432. The molecule has a hydroxy decanoate chain (substituted on C3 of ring A) where part of it was exposed to the solvent, and the rest entered a small groove in the active site, adding extra binding interactions (Figure 7).

**Figure 8:**
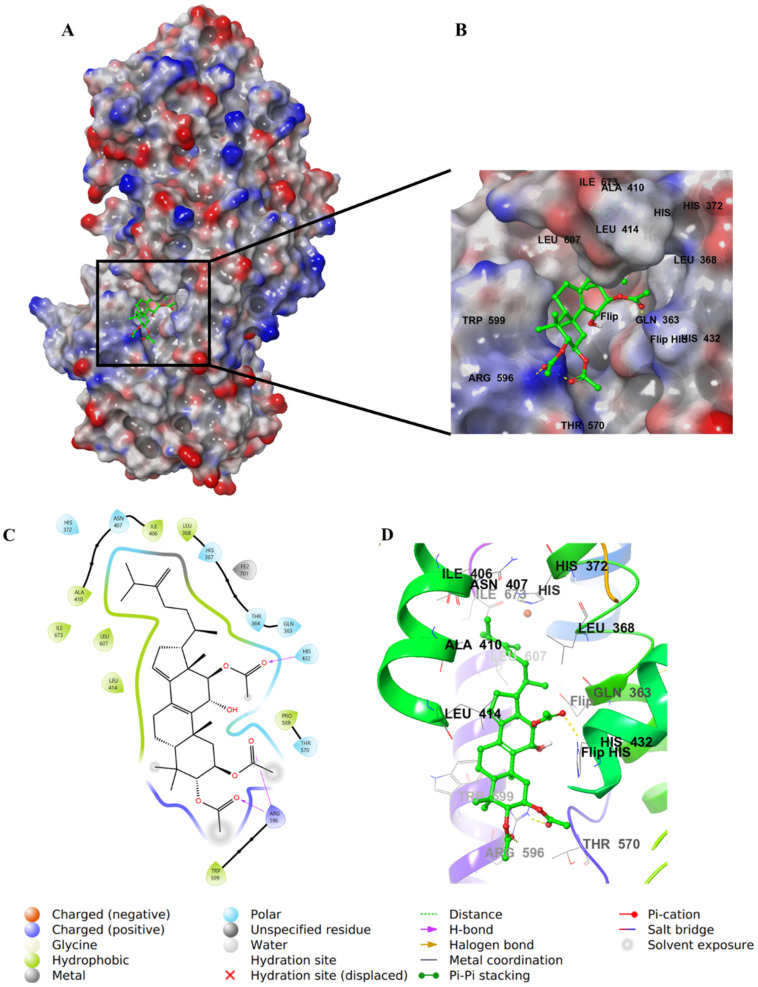
Molecular docking of integracide H (**5**) in stable human 5-LOX (PDB: 6N2W). (**A**) Molecular surface representation with solid style and electrostatic potential color scheme of red, white, and blue (min −0.3, max +0.3); (**B**) Zoomed look of integracide H (**5**) in the active site of 5-LOX in molecular surface; (**C**) 2D representation of binding interactions of integracide H (**5**) with amino acid residues in the active site within 4 Å distance; (**D**) 3D representation of integracide H (**5**) in green color within the active site of 5-LOX. The H–bond interactions are displayed in yellow dotted lines.

Integracide H (**5**) contains two acetyloxy substituents on C2 and C3 of ring A and is involved in H–bond interactions with Arg596. However, the two groups were partially solvent-exposed (Figure 8).

**Figure 9:**
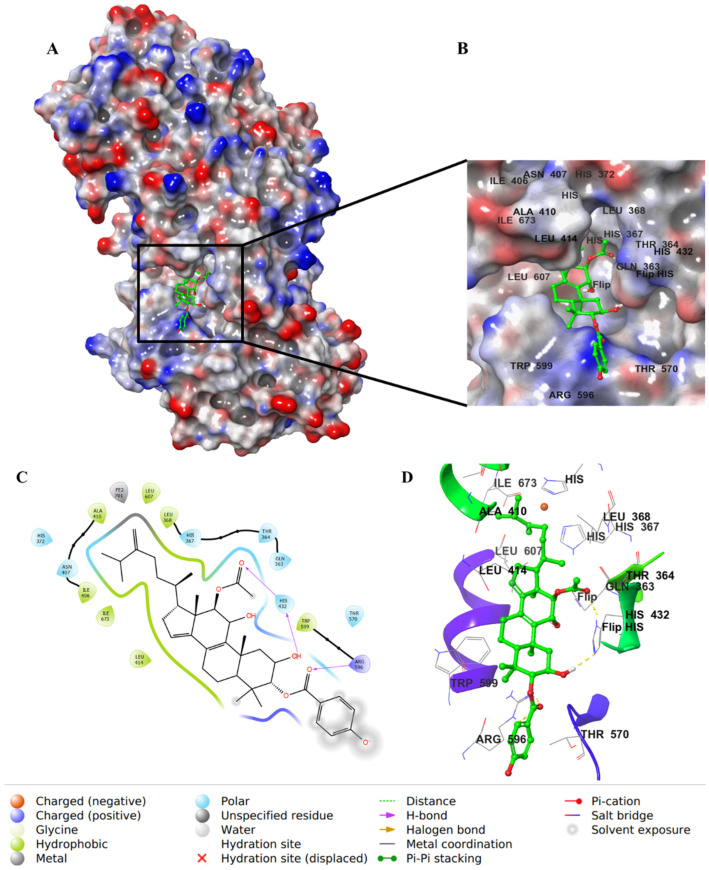
Molecular docking of integracide J (**6**) in stable human 5-LOX (PDB: 6N2W). (**A**) Molecular surface representation with solid style and electrostatic potential color scheme of red, white, and blue (min −0.3, max +0.3); (**B**) Zoomed look of integracide J (**6**) in the active site of 5-LOX in molecular surface; (**C**) 2D representation of binding interactions of integracide J (**6**) with amino acid residues in the active site within 4Å distance; (**D**) 3D representation of integracide J (**6**) in green color within the active site of 5-LOX. The H–bond interactions are displayed in yellow dotted lines.

For integracide J (**6**), it has a *p*-hydroxybenzoyloxy substitution on C3 of ring A instead of the long chain present in **4**. The C=O of the acetyloxy group on C12 and -OH on C2 interacted with His432 through H–bonds. Moreover, the benzoyl C=O formed an H–bond with Arg596; however, the phenolic OH group was exposed to the solvent and did not involve any type of binding with the protein (Figure 9).

**Figure 10:**
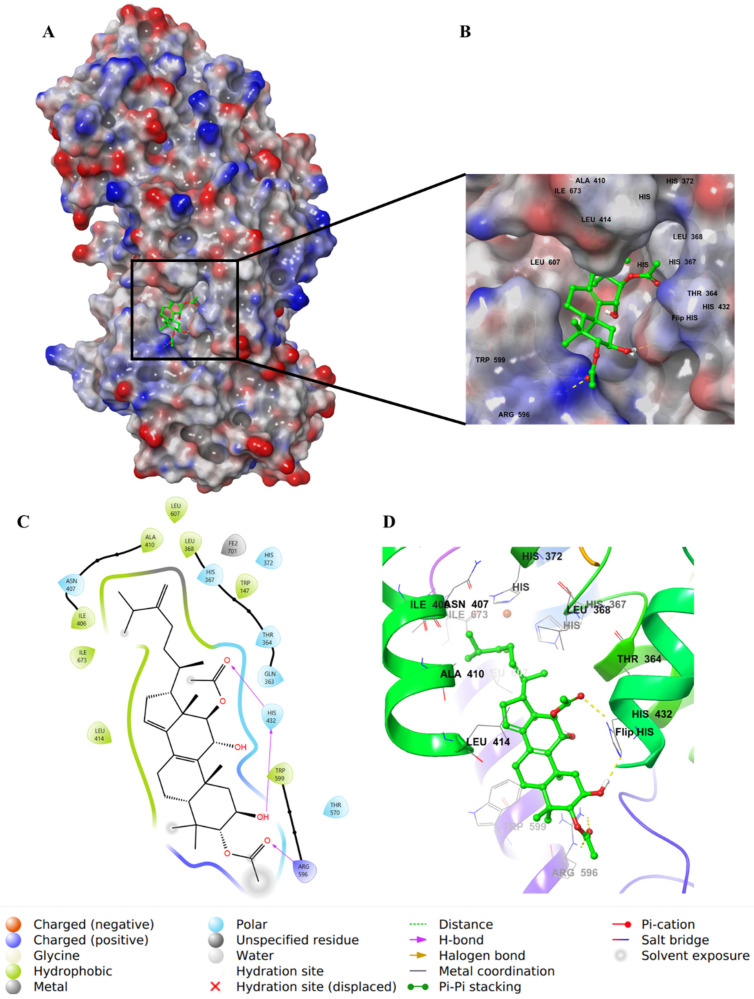
Molecular docking of integracide F (**3**) in stable human 5-LOX (PDB: 6N2W). (**A**) Molecular surface representation with solid style and electrostatic potential color scheme of red, white, and blue (min −0.3, max +0.3); (**B**) Zoomed look of integracide F (**3**) in the active site of 5-LOX in molecular surface; (**C**) 2D representation of binding interactions of integracide F (**3**) with amino acid residues in the active site within 4 Å distance; (**D**) 3D representation of integracide F (**3**) in green color within the active site of 5-LOX. The H–bond interactions are displayed in yellow dotted lines.

Integracide F (**3**) has one acetyloxy group on C3 of ring A. The molecule showed similar H–bond interactions with His432 and Arg596 (Figure 10).

**Figure 11:**
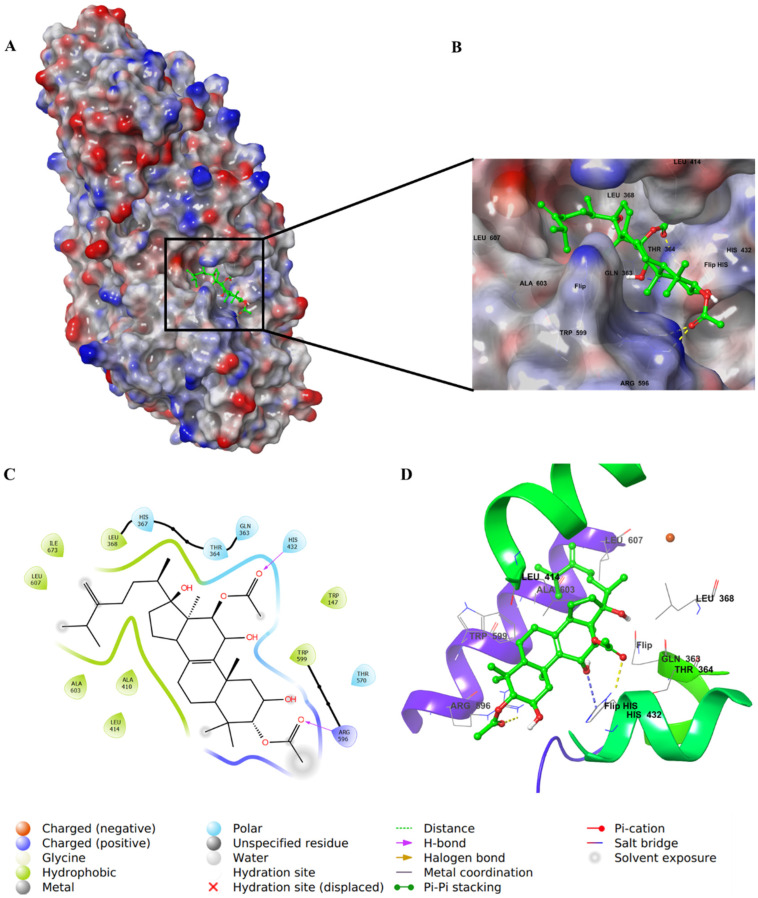
Molecular docking of integracide L (**1**) in stable human 5-LOX (PDB: 6N2W). (**A**) Molecular surface representation with solid style and electrostatic potential color scheme of red, white, and blue (min −0.3, max +0.3); (**B**) Zoomed look of integracide L (**1**) in the active site of 5-LOX in molecular surface; (**C**) 2D representation of binding interactions of integracide L with amino acid residues in the active site within 4 Å distance; (**D**) 3D representation of integracide L (**1**) in green color within the active site of 5-LOX. The H–bond and aromatic-hydrogen interactions are in yellow and purple dotted lines, respectively.

Integracide L (**1**) has a saturated cyclopenty ring and OH group on C17, which are not present in any other integracide analogs. These modifications may not be favored, directing the R group away from the hydrophobic pocket and being more exposed to the solvent. This change may influence the binding affinity and increase the gscore and IC_50_ for this compound (Table 2 and Figure 4). The acetyloxy groups on rings A and C formed H–bonds with Arg596 and His432, respectively (Figure 11).

**Figure 12:**
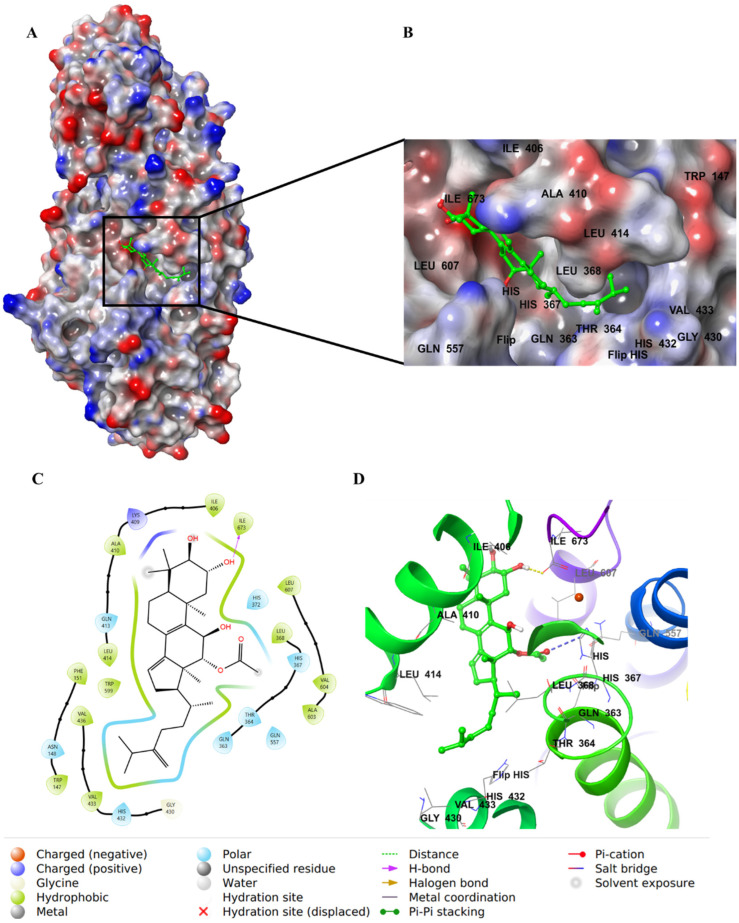
Molecular docking of integracide B (**2**) in stable human 5-LOX (PDB: 6N2W). (**A**) Molecular surface representation with solid style and electrostatic potential color scheme of red, white, and blue (min −0.3, max +0.3); (**B**) Zoomed look of integracide B in the active site of 5-LOX in molecular surface; (**C**) 2D representation of binding interactions of integracide B with amino acid residues in the active site within 4 Å distance; (**D**) 3D representation of integracide B in green color within the active site of 5-LOX. The H–bond and aromatic-hydrogen interactions are in yellow and purple dotted lines, respectively.

Integracide B (**2**) was bound to the active site in a different mode, opposite to what has been noted with other integracides (Figure 12). The presence of free OH groups on ring A could contribute to its low binding affinity, illustrated by the gscore of −5.051 kcal/mol and IC_50_ value of 3.97 μM, among other integracides (Table 2).

**Figure 13:**
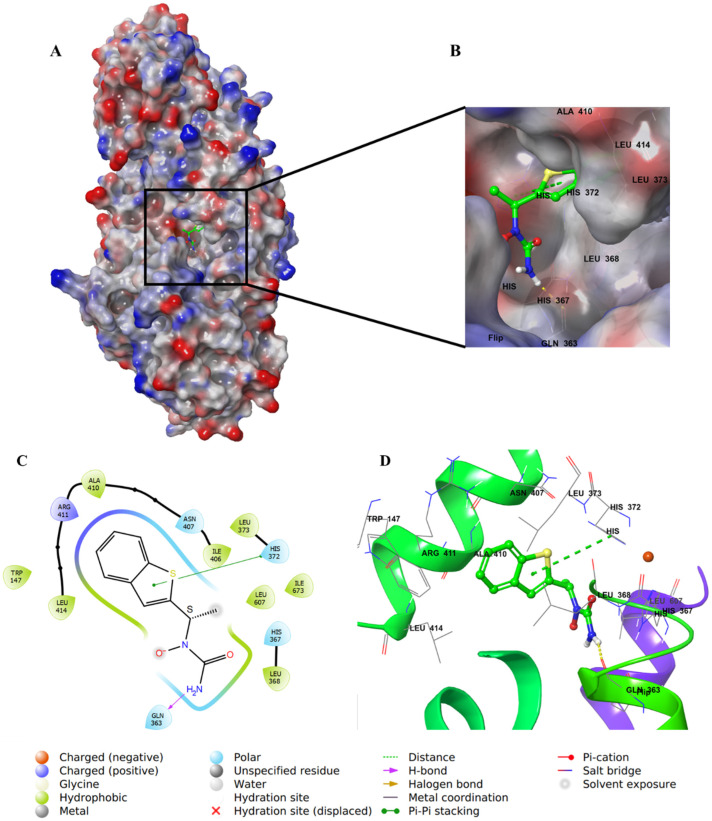
Molecular docking of zileuton in stable human 5-LOX (PDB: 6N2W). (**A**) Molecular surface representation with solid style and electrostatic potential color scheme of red, white, and blue (min −0.3, max +0.3); (**B**) Zoomed image of zileuton in the active site of 5-LOX in molecular surface; (**C**) 2D representation of binding interactions of zileuton with amino acid residues in the active site within 4 Å distance; (**D**) 3D representation of zileuton in green color within the active site of 5-LOX. The H–bond and π–π staking interactions are in yellow and green dotted lines, respectively.

**Figure 14:**
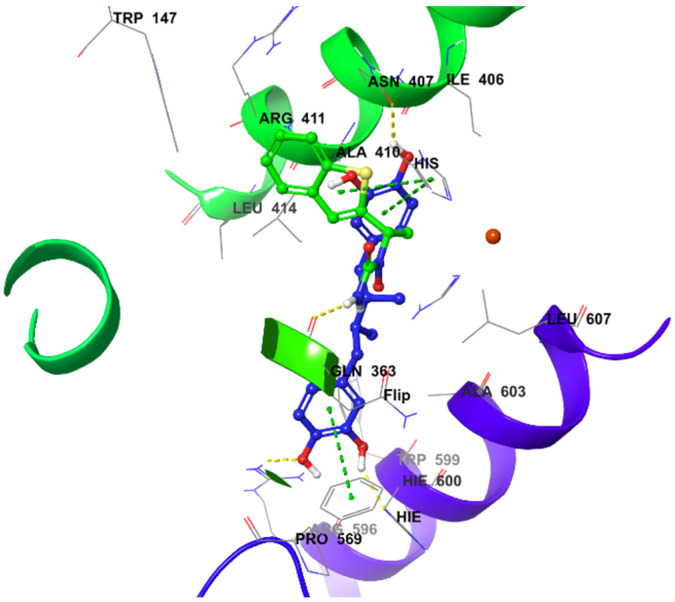
The 3D representation of zileuton-NDGA overlay in the active site of 5-LOX (PDB: 6N2W).

The positive control inhibitor, zileuton, was also docked in the 5-LOX active site to investigate its binding mode. The amide-NH_2_ formed an H–bond with Gln363, whereas the thiophene ring formed a π–π interaction with the imidazole ring in His372 (Figure 13), similar to what was observed for NDGA (Figure 14). Although zileuton produced the highest potency in the biochemical assay (IC_50_ 1.17 μM), little correlation was observed with its gscore (−4.766 kcal/mol), and it ranked last among the other tested compounds (Table 2).

**Figure 15:**
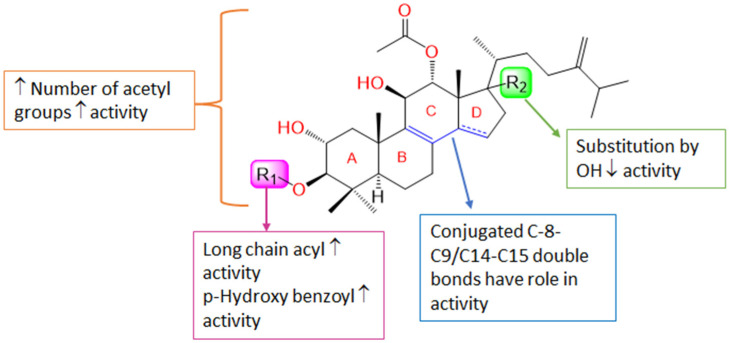
Structure–activity relationship of integracides. ↓: decrease; ↑: increase.

Accordingly, it was noted that the substitution pattern of the intergracides’ terpenoid framework might influence the efficacy. Substitution at C-3 by long-chain fatty acid acyl as in **4** or *p*-hydroxy benzoyl as in **6** was found to elevate the activity. Additionally, increasing the number of acetyl groups raised the activity, as well as the conjugated C_8_-C_9_-C_14_-C_15,_ which may have a role in the activity (Figure 15). Further, the lacking a C14-C15 double bond and the presence of the C17-OH group substitution minimized the efficacy.

## 4. Material and Methods

### 4.1. General Experimental Procedures

Optical rotation was estimated using a 341LC Perkin-Elmer polarimeter (Perkin-Elmer, Waltham, MA, USA). UV and IR were assessed employing Shimadzu UV/VIS 1601 and IR-400 spectrophotometers (Shimadzu, Kyoto, Japan), respectively. Orbitrap LTQ (ThermoFinnigan, Bremen, Germany) and 850 INOVA BRUKER (Bruker BioSpin, Billerica, MA, USA) were utilized for HRESIMS and NMR measuring. The chromatographic investigation was achieved utilizing SiO_2_60/Sephadex LH-20/RP-18, in addition to a 6-mL RP-18 LiChrolut extraction tube (Merck/Darmstadt/Germany) [20,30]. TLC was carried out on F_254_ SiO_2_60 TLC plates while Linoleic acid, 5-LOX kits, and zileuton were secured by Sigma–Aldrich (Sigma-Aldrich, St. Louis, MO, USA).

### 4.2. Cultivation of Fungal Material

The earlier separated and identified *Fusarium* sp. (FS No. MAR2014) was cultivated in 20 Erlenmeyer flasks (1 L each) as stated formerly [20,30].

### 4.3. Extraction and Isolation

The culture extraction was carried out utilizing EtOAc and was concentrated by a vacuum. Subsequently, mixing the extract with H_2_O (300 mL) and partitioning among *n*-hexane and MeOH (90%) were performed. The MeOH extract (6.9 g) was submitted to the Sephadex LH-20 column chromatography (CC, MeOH/CHCl_3_ 30/70) to result in eight sub-fractions: FS.1–FS.8. Sub-fraction FS.3 (311 mg) was separated on SiO_2_ CC (EtOAc/*n*-hexane 2/98–30/70) to yield **1**, which was submitted to RP-18 CC (H_2_O/MeOH) to obtain **1** (4.8 mg). Sub-fraction FS.4 (465 mg) SiO_2_ CC (EtOAc/*n*-hexane 5:95–30:70) afforded **2**, following that of RP-18 LiChrolut (acetonitrile/H_2_O gradient) to yield **2** (5.1 mg). Sub-fraction FS.5 (970 mg) SiO_2_ CC (EtOAc/*n*-hexane 3/97–20/80) following this RP18 CC (MeOH/H_2_O gradient) produced **3** (27.4 mg).

#### NMR Spectral Data of Integracide L (**1**)

White amorphous powder; [α]D +19.3 (*c* 0.2, MeOH); UV λ*_max_* (MeOH) (log *ε*): 239 (3.27) nm; IR (KBr) *ν_max_* 3439, 1729, 1663, 879 cm^−1^; NMR data: see Table 1; HRESIMS *m*/*z* 575.3954 (calcd for C_34_H_55_O_7_, 575.3948 [M + H]^+^).

### 4.4. 5-Lipoxygenase Inhibitory Assay

5-LOI of integracides (**1**–**6**, Conc. 0.001, 0.01, 1.0, and 10 µM) was examined as priorly stated, utilizing zileuton as the positive control [2,48,49,50,51,52].

### 4.5. Statistical Analysis

IC_50_s were estimated by regression analysis (GraphPad-InStat 3, GraphPad Software, San Diego, CA, USA). The % 5-LOX inhibition of and IC_50_s are listed as mean-values ±SD. Statistical significance was analyzed among the samples by one-way-ANOVA and subsequently by Tukey–Kramer test (*p* < 0.005).

### 4.6. Molecular Docking Study

#### 4.6.1. Ligand and Protein Preparation

The docking study was performed with the Schrodinger program (Schrödinger Release 2021-4: LigPrep, Schrödinger, LLC, New York, NY, USA, 2021). The crystal structure of stable 5-LOX complexed with the NDGA inhibitor was downloaded from the protein data bank (PDB; ID: 6N2W). The protein was prepared using the “Protein Preparation Wizard” tool in Maestro software, where the missing hydrogens were added to the residues, the metal ionization state was corrected, and the water molecules > 5 Å from protein residues were deleted. The protein contained two chains: A and B. Chain B was complexed with the inhibitor; therefore, it was chosen to perform the docking study. The protein was then refined by predicting the pKa of the ionizable residues using PROPKA and water molecules > 3 Å (not involved in the water bridge), which were removed [36]. Additionally, the proteins containing Fe^2+^ coordinates with His residues were in the active site. The protein-Fe^2+^ bonds were deleted, and finally, the minimization of the protein was applied using the OPLS4 force field. All ligands were prepared before docking using the “LigPrep” tool [53]. The 2D structures of the ligands were converted to 3D and energy-minimized using the OPLS3 force field. The hydrogens were added, and all possible ionization states and tautomeric forms were created at a pH of 7.0 ± 0.2 by Epik; a desalt option was also chosen. The H–bonds were optimized by predicting the pKa of ionizable groups using PROPKA [36].

#### 4.6.2. Grid Generation and Molecular Docking

To perform the docking, a grid box was generated around the active site of stable 5-LOX (PDB: 6N2W) containing the co-crystallized inhibitor NDGA and using Glide’s “Receptor-Grid-Generation” tool in Schrödinger suite [39]. All study ligands were docked inside the grid box with an extra precision (XP) protocol, and all other parameters were set to default [41]. The non-polar atoms were set for the VdW radii scaling factor by 1.0, and the partial charge cut-off was 0.25. Finally, the “Ligand Docking” tool was implemented for docking [40]. To validate the docking method, the co-crystallized inhibitor was re-docked inside the grid box and evaluated.

## 5. Conclusions

A new tetracyclic triterpenoid, integracide L (**1**) and two metabolites (**2** and **3**), were purified from *Fusarium* sp. (FS No. MAR2014). Their specification was achieved with the assistance of extensive spectral analyses. Compounds **1**–**6** demonstrated a noticeable 5-LOX inhibition potential. The docking study of integracides illustrated their binding mode in the active site with possible amino acid interactions, which could explain their inhibitory activity.

These findings may foster more inspection of the possible usage of integracides as 5-LOX inhibitors and draw further interest to the synthesis of structure-similar analogs with enhanced 5-LOX inhibition capacity. Moreover, additional in vivo and mechanistic investigations are needed.

## Figures and Tables

**Table 1:** NMR spectral data of compound **1** (DMSO-*d*_6_, 850 and 214 MHz).

No.	δ_H_ [mult., *J* (Hz)]	δ_C_ (mult.)	HMBC
1	2.65 m 1.11 m	42.6 CH_2_	2, 3, 5
2	3.73 dd (10.3, 4.2)	66.8 CH	1, 3, 5
3	3.62 d (10.3)	88.0 CH	2, 4, 29, 30, 33
4	-	39.2 C	-
5	1.03 dd (12.8, 3.4)	51.1 CH	3, 4, 7, 10, 18
6	1.66 m 1.52 m	18.0 CH_2_	5, 7, 8
7	2.21 m 1.46 m	26.8 CH_2_	5, 8, 9
8	-	127.4 C	-
9	-	143.5 C	-
10	-	38.5 C	-
11	4.25 brs	67.9 CH	8, 9, 12
12	5.00 d (3.4)	78.3 CH	9, 11, 13, 14, 19, 31
13	-	47.6 C	-
14	2.06 t (6.2)	39.8 CH	-
15	1.68 m 1.45 m	31.3 CH_2_	8, 13, 14, 17
16	2.28 m 2.18 m	33.5 CH_2_	14, 15, 17
17	-	86.0 C	13, 16, 22
18	1.20 s	22.1 CH_3_	1, 5, 9, 10
19	0.61 s	13.2 CH_3_	12, 13, 14, 17
20	1.67 m	33.0 CH	17, 21, 23
21	1.22 d (6.3)	21.8 CH_3_	17, 20, 22
22	2.08 m 1.87 m	31.7 CH_2_	21, 24
23	2.41 m 1.81 m	35.0 CH_2_	24, 25, 28
24	-	155.4 C	-
25	2.19 m	32.3 CH	23, 24, 26, 27
26	0.98 d (6.8)	21.6 CH_3_	24, 25, 27
27	0.96 d (6.8)	21.5 CH_3_	24, 25, 26
28	4.70 brs 4.66 brs	106.9 CH_2_	23, 24, 25
29	0.73 s	17.9 CH_3_	3, 4, 5, 30
30	0.91 s	28.5 CH_3_	3, 4, 5, 29
31	-	169.5 C	-
32	1.98 s	20.6 CH_3_	31
33	-	170.1 C	-
34	2.05 s	21.0 CH_3_	33
2-OH	5.30 brs	-	1, 2, 3
11-OH	5.21 d (6.8)	-	9, 11
17-OH	5.39 s	-	13, 17

**Table 2:** Docking results of integracides with stable 5-LOX (PDB: 6N2W).

Compound	XP Gscore	Glide Gscore	Glide Emodel
NDGA	−7.856	−7.856	−51.600
Integracide G (**4**)	−6.708	−6.708	−66.318
Integracide F (**3**)	−6.453	−6.453	−47.304
Integracide J (**6**)	−6.169	−6.169	−47.646
Integracide H (**5**)	−5.221	−5.221	−46.847
Integracide L (**1**)	−5.109	−5.109	−34.117
Integracide B (**2**)	−5.051	−5.051	−35.498
Zileuton	−4.766	−4.766	−31.899

## Data Availability

Not applicable.

## Supplementary Materials

The following supporting information can be downloaded at: https://www.mdpi.com/article/10.3390/life12122095/s1. 1H and 13C NMR data of integracides (**2**–**6**).
