# Supplementary material for: Integracides: Tetracyclic Triterpenoids from Fusarium sp.—Their 5-Lipoxygenase Inhibitory Potential and Structure–Activity Relation Using In Vitro and Molecular Docking Studies
Source: Life (Basel). 2022 Dec 13;12(12):2095. doi: 10.3390/life12122095 (PMC9782297; doi:10.3390/life12122095)
Supplement: Supplementary file 1 [file life-12-02095-s001.zip › life-2069020-supplementary.pdf]

life

Integracides: Tetracyclic Triterpenoids from *Fusarium* sp. – Their 5-Lipoxygenase Inhibitory Potential and Structure-activity Relation Using In Vitro and Molecular Docking Studies

Maan T. Khayat [1,*], Khadijah A. Mohammad [1], Gamal A. Mohamed [2], Martin K. Safo [3], Sabrin R. M. Ibrahim [4,5]

[1] Department of Pharmaceutical Chemistry, Faculty of Pharmacy, King Abdulaziz University, Jeddah 21589, Saudi Arabia

[2] Department of Natural Products and Alternative Medicine, Faculty of Pharmacy, King Abdulaziz University, Jeddah 21589, Saudi Arabia

[3] Department of Medicinal Chemistry, School of Pharmacy, Virginia Commonwealth University, Richmond, VA 23219, USA

[4] Department of Chemistry, Preparatory Year Program, Batterjee Medical College, Jeddah 21442, Saudi Arabia

[5] Department of Pharmacognosy, Faculty of Pharmacy, Assiut University, Assiut 71526, Egypt

* Correspondence: Mkhayat@kau.edu.sa; Tel.: +966 555543053

Integracide B (**2**): ^{1}H NMR (DMSO-d_6, 850 MHz): δ_H 2.26 (dd, *J* = 12.8, 4.3 Hz, H1), 3.74 (td, *J* = 10.2, 4.3 Hz, H2), 3.57 (d, *J* = 10.2 Hz, H3), 1.07 (dd, 12.7, 2.6 Hz, H5), 1.67 (m, H6A), 1.56 (m, H6B), 2.30 (m, H7A), 2.19 (m, H7B), 4.07 (d, *J* = 2.6 Hz, H11), 4.95 (brs, H12), 5.50 (brs, H15), 2.33 (m, H16A), 1.99 (m, H16B), 1.84 (dt, *J* = 11.1, 8.5 Hz, H17), 1.19 (s, H18), 0.99 (s, H19), 1.58 (m, H20), 0.84 (d, *J* = 6.0 Hz, H21), 1.51 (m, H22A), 1.10 (m, H22B), 2.05 (m, H23A), 1.87 (m, H23B), 2.18 (m, H25), 0.97 (d, *J* = 6.6 Hz, H26), 0.97 (d, *J* = 6.6 Hz, H27), 4.70 (brs, H28A), 4.65 (brs, H28B), 0.75 (s, H29), 0.95 (s, H30), 2.00 (s, H32), 5.31 (brs, 2OH), 5.39 (d d, *J* = 6.0 Hz, 11OH); ^{13}C NMR (DMSO-d_6, 214 MHz): δ_C 43.4 (C1), 66.8 (C2), 88.0 (C3), 39.5 (C4), 50.3 (C5), 17.7 (C6), 26.2 (C7), 123.8 (C8), 139.4 (C9), 37.2 (C10), 67.4 (C11), 77.7 (C12), 46.4 (C13), 147.0 (C14), 120.2 (C15), 34.8 (C16), 48.5 (C17), 22.6 (C18), 16.6 (C19), 32.7 (C20), 17.9 (C21), 33.9 (C22), 30.4 (C23), 155.7 (C24), 33.1 (C25), 21.8 (C26), 21.7 (C27), 106.5 (C28), 17.4 (C29), 28.6 (C30), 169.8 (C31), 21.0 (C32).

Integracide F (**3**): ^{1}H NMR (CDCl$_3$, 850 MHz): δ_H 2.23 (m, H1), 3.76 (ddd, *J* = 11.4, 10.1, 4.0 Hz, H2, H2), 3.59 (d, *J* = 10.1 Hz, H3), 1.70 (dd, *J* = 11.9, 3.1 Hz, H5), 1.69 (m, H6A), 1.60 (m, H6B), 2.32 (m, H7A), 2.20 (m, H7B), 4.09 (brs. H11), 4.97 (d, *J* = 2.1 Hz, H12), 5.52 (brt, *J* = 2.5 Hz, H15), 2.39 (m, H16A), 2.00 (m, H16B), 1.83 (dt, *J* = 10.3, 7.3 Hz, H17), 1.21(s, H18), 0.98 (s, H19), 1.60 (m, H20), 0.86 (d, *J* = 6.2 Hz, H21), 1.54 (m, H22A), 1.14 (m, H22B), 2.05 (m, H23A), 1.88 (m, H23B), 2.19 (m, H25), 0.99 (d, *J* = 6.5 Hz, H26), 1.00 (d, *J* = 6.5 Hz, H27), 4.71 (brs, H28A), 4.66 (brs, H28B), 0.78 (s, H29), 0.96 (s, H30), 2.05 (s, H32), 2.01 (s, H34), 5.32 (brs, 2OH), 5.38 (brs, 11OH); ^{13}C NMR (CDCl$_3$, 214 MHz): δ_C 43.8 (C1), 67.3 (C2), 88.5 (C3), 39.5 (C4), 50.7 (C5), 18.2 (C6), 26.6 (C7), 124.2 (C8), 139.9 (C9), 37.7 (C10), 67.9 (C11), 78.2 (C12), 46.9 (C13), 147.5 (C14), 120.7 (C15),

35.3 (C16), 49.0 (C17), 23.1 (C18), 17.0 (C19), 33.2 (C20), 18.3 (C21), 34.4 (C22), 30.9 (C23), 156.2 (C24), 33.5 (C25), 22.2 (C26), 22.1 (C27), 107.0 (C28), 17.9 (C29), 29.1 (C30), 170.5 (C31), 21.4 (C32), 170.3 (C33), 21.4 (C34).

Integracide G (**4**): ^{1}H NMR ($CDCl_3$, 850 MHz): δ_H 2.27 (m, H1A), 1.06 (m, H1B), 3.76 (ddd, *J* = 11.2, 10.0, 4.0 Hz, H2), 3.59 (d, *J* = 9.5 Hz, H3), 1.12 (dd, *J* = 11.6, 3.2 Hz, H5), 1.69 (m, H6A), 1.60 (m, H6B), 2.32 (m, 7A), 2.08 (m, H7B), 4.10 (brs, H11), 4.98 (brd, *J* = 2.0 Hz, H12), 5.52 (brt, *J* = 2.0 Hz, H15), 2.35 (m, H16A), 2.02 (m, H16B), 1.87 (dt, *J* = 10.6, 7.5 Hz, H17), 1.21 (s, H18), 0.98 (s, H19), 1.61 (m, H20), 0.86 (d, *J* = 6.3 Hz, H21), 1.52 (m, H22A), 1.15 (m, H22B), 2.08 (m, H23A), 1.89 (m, H23B),2.18 (m, H25), 0.99 (d, *J* = 6.5 Hz, H26), 1.00 (d, *J* = 6.5 Hz, H27), 4.72 (brs, H28A), 4.67 (brs, H28B), 0.78 (s, H29), 0.95 (s, H30), 1.98 (s, H32), 2.32 (t, J = 7.2 Hz, H34), 1.17 (m, H35), 1.26–1.22 (m, H36-39), 1.61 (m, H40), 1.25 (m, H41), 3.35 (t, J = 6.8 Hz, H42), 5.33 (brs, 2OH), 5.38 (brs, 11OH); ^{13}C NMR ($CDCl_3$, 214 MHz): δ_C 43.8 (C1), 67.3 (C2), 88.4 (C3), 39.4 (C4), 50.8 (C5), 18.1 (C6), 26.6 (C7), 124.7 (C8), 139.6 (C9), 37.7 (C10), 67.8 (C11), 78.4 (C12), 46.6 (C13), 147.1 (C14), 120.6 (C15), 35.4 (C16), 49.1 (C17), 23.1 (C18), 17.0 (C19), 33.4 (C20), 18.3 (C21), 34.4 (C22), 31.7 (C23), 156.5 (C24), 33.6 (C25), 22.2 (C26), 22.1 (C27), 107.0 (C28), 17.9 (C29), 29.1 (C30), 170.3 (C31), 21.4 (C32), 170.6 (C33), 34.0 (C34), 23.7 (C35), 29.5–29.1 (C36-39), 24.8 (C40), 30.9 (C41), 63.1 (C42).

Integracide H (**5**): ^{1}H NMR (DMSO-d_6, 850 MHz): δ_H 5.50 (brs, H15), 4.95 (brs, H12), 4.70 (brs, H28A), 4.65 (brs, H28B), 4.08 (brs, H11), 3.86 (td, *J* = 10.6, 4.3 Hz, H2), 3.58 (d, *J* = 10.6 Hz, H3), 2.35 (m, H16A), 2.28 (m, H1A), 2.32 (m, H7A), 2.21 (m, H7B), 2.20 (m, H25), 2.08 (m, H23A), 2.00 (m, H16B), 1.95 (s, H32), 1.89 (s, H34), 1.89 (s, H36), 1.87 (m, H23B), 1.85 (dt, *J* = 11.6, 8.5 Hz, H17), 1.82 (m, H6A), 1.63 (m, H-6B), 1.58 (m, H20), 1.51 (m, H22A), 1.20 (s, H18), 1.11 (dd, *J* = 12.6, 2.8 Hz, H5), 1.02 (m, H22B), 1.01 (m, H1B), 0.98 (s, H19), 0.97 (d, *J* = 6.4 Hz, H27), 0.96 (d, *J* = 6.4 Hz, H26), 0.95 (s, H30), 0.84 (d, *J* = 6.4 Hz, H21), 0.74 (s, H29); ^{13}C NMR (DMSO-d_6, 214 MHz): δ_C 43.4 (C1), 66.8 (C2), 88.1 (C3), 39.5 (C4), 50.2 (C5), 17.8 (C6), 26.4 (C7), 123.9 (C8), 139.4 (C9), 39.9 (C10), 67.7 (C11), 77.8 (C12), 46.4 (C13), 147.1 (C14), 120.3 (C15), 35.0 (C16), 48.6 (C17), 22.7 (C18), 16.6 (C19), 32.8 (C20), 17.9 (C21), 33.9 (C22), 30.4 (C23), 155.3 (C24), 172.1 (C33,35), 169.9 (C31), 33.1 (C25), 21.8 (C26), 21.7 (C27), 106.6 (C28), 17.5 (C29), 28.5 (C30), 21.0 (C32), 21.1 (C34,36).

Integracide J (**6**): ^{1}H NMR (DMSO-d_6, 850 MHz): δ_H 2.26 (m, H1A), 1.06 (m, H1B), 3.74 (td, *J* = 10.4, 4.3 Hz, H2), 3.57 (d, *J* = 10.4 Hz, H3), 1.11 (dd, *J* = 12.8, 2.8 Hz, H5), 1.68 (m, H6A), 1.60 (m, H6B), 2.32 (m, H7A), 2.21 (m, H7B), 4.07 (d, *J* = 2.4 Hz, H11), 4.95 (d, *J* = 1.7 Hz, H12), 5.50 (brs, H15), 2.38 (m, H16A), 2.01 (m, H16B), 1.85 (dt, *J* = 11.4, 8.5 Hz, H17), 1.19 (s, H18), 0.98 (s, H19), 1.58 (m, H20), 0.84 (d, *J* = 6.4 Hz, H21), 1.65 (m, H22A), 1.12 (m, H22B), 2.07 (m, H23A), 1.88 (m, H23B), 2.21 (m, H25), 0.96 (d, *J* = 6.6 Hz, H26), 0.99 (d, *J* = 6.6 Hz, H27), 4.70 (d, *J* = 0.9 Hz, H28A), 4.65 (d, *J* = 0.9 Hz, H28B), 0.75 (s, H29), 0.95 (s, H30), 1.96 (s, H32), 7.00 (d, *J* = 8.5 Hz, H35,39), 6.61 (d, *J* = 8.5 Hz, H36,38), 5.31 (s, 2OH), 5.39 (d, *J* = 6.0 Hz, 11OH), 9.13 (s, 37OH); ^{13}C NMR (DMSO-d_6, 214 MHz): δ_C 43.3 (C1), 66.8 (C2), 88.0 (C3), 39.5 (C4), 50.2 (C5), 17.7 (C6), 26.2 (C7), 123.8 (C8), 139.4 (C9), 37.2 (C10), 67.4 (C11), 77.7 (C12), 46.4 (C13), 147.0 (C14), 120.2 (C15), 34.8 (C16), 48.5 (C17), 22.6 (C18), 16.5 (C19), 32.7 (C20), 17.9 (C21), 33.9 (C22), 31.3 (C23), 155.7 (C24), 33.1 (C25), 21.8 (C26), 21.7 (C27), 106.5 (C28), 17.4 (C29), 28.5 (C30), 169.8 (C31), 21.0 (C32), 170.1 (C33), 127.9 (C34), 130.1 (C35,39), 114.8 (C36,38), 155.7 (C39).
